# Impact resistance of composite magnetic metamaterials

**DOI:** 10.1038/s41598-019-40610-w

**Published:** 2019-03-08

**Authors:** Krzysztof K. Dudek, Wiktor Wolak, Ruben Gatt, Joseph N. Grima

**Affiliations:** 1 Institute of Physics, University of Zielona Gora, ul. Szafrana 4a, 65-069 Zielona Gora, Poland; 2 Metamaterials Unit, Faculty of Science, University of Malta, Msida, MSD 2080 Malta; 3 Department of Chemistry, Faculty of Science, University of Malta, Msida, MSD 2080 Malta

## Abstract

In this work, through numerical studies, we show the possibility of designing composites in a form of magneto-mechanical metamaterials which are capable of exhibiting an enhanced impact resistance in comparison to their non-magnetic counterparts. We also show that it is possible to control the impact resistance of the system solely by means of the magnitude of the magnetic moment associated with magnetic inclusions inserted into the system as well as through the way how magnetic inclusions are distributed within the structure. The latter result is particularly interesting as in this work we show that through the appropriate distribution of magnetic inclusions it is possible to minimise the force that is being transferred to an object through the protective mechanical metamaterial. It is also suggested that the concept proposed in this work can be implemented in the case of already existing protective devices such as military-related protective devices and car bumpers in order to increase their efficiency.

## Introduction

Everyday activities such as traveling by car and playing different sports may put our health at risk due to potential injuries which we may suffer as a result of collisions with an external object. To ensure our safety, we often seek different protective devices which could minimise the severity of potential injuries. In view of this, in the last decades, the importance of studies related to impact and indentation resistance of materials^1–10^ has been growing rapidly with a particular emphasis on commercial protective devices which are utilised by the modern society on the daily basis. Some prime examples of such devices include helmets and car bumpers^11–13^ with their sole purpose being to withstand the impact of a collision with an external body. In addition to everyday protective devices, the studies related to the enhanced impact and indentation resistance play a big role in the military industry^5–7^ where they contribute to the design of different types of shielding devices devoted amongst others to vehicles and personal protection equipment. In order to design such a variety of devices it is necessary to use materials which are light and can be tailor-made to exhibit a desired type of mechanical response depending on the particular application. Such requirement is met by some of the mechanical metamaterials and composites that thanks to their properties can amongst others exhibit an enhanced impact resistance.

Mechanical metamaterials^14^ are commonly defined as a class of systems, which exhibit unusual mechanical properties as a result of their design rather than their chemical composition. Mechanical metamaterials include systems that exhibit negative Poisson’s ratio (auxetic behaviour)^15–24^, negative stiffness^25–30^, negative compressibility^31–36^ and negative thermal expansion^37–42^. From the point of view of the minimisation of the damage caused by a collision of a material with an external body, the most important of these studies seem to be those related to auxetic behaviour. This stems from the fact that negative Poisson’s ratio enables the system to exhibit an enhanced indentation resistance due to the fact that in auxetics, the material tends to flow towards the point of impact rather than away from it (as in the case of non-auxetic materials).

Similarly to mechanical metamaterials, enhanced impact resistance can also be exhibited by a variety of composites^43–50^ with one example being composites in the form of laminates. These composites consist of a number of topologically different materials exhibiting different mechanical properties. For such systems, the energy associated with the impact can be predominantly absorbed by specific parts of the structure which allows to minimise the damage inflicted on the other part of the structure. Impact resistant composites can also be designed through the use of inclusions inserted into the system^51–54^. In theory, it could even happen that one would design the composite with superior impact resistant properties through the introduction of appropriate inclusions into an otherwise non-composite system. This in turn is very interesting as it means that one can consider the use of inclusions in the case of otherwise non-composite mechanical metamaterials in order to make them become composites. Such inclusions can in general be of an arbitrary form but it seems that one of the most promising candidates to investigate this concept are magnetic inclusions. This stems from the fact that the use of magnetic inclusions in mechanical metamaterials^30,55–60^ has already been reported to allow to control their behaviour and mechanical properties. However, it should be noted that this concept is still in its infancy and there are many aspects related to these systems which remain to be thoroughly investigated. One such aspect is the distribution of magnetic inclusions within a mechanical metamaterial. Amongst other reasons, this stems from the fact that through the non-uniform distribution of magnetic inclusions within the structure (or distribution of magnetic inclusions associated with magnetic moments of a different magnitude) it is possible to construct a mechanical metamaterial which despite not looking like a composite laminate in terms of geometry could be expected to exhibit a very similar behaviour.

In view of all this, in this study an attempt will be made to design a magneto-mechanical metamaterial which despite not exhibiting unusual mechanical properties in the direction associated with the collision manifests a superior indentation resistance in comparison to its conventional non-magnetic counterpart. An attempt will also be made in order to show that an insertion of non-uniformly distributed magnetic inclusions into an otherwise uniformly designed mechanical metamaterial, can make it act like a composite composed of a number of topologically different materials. Such an approach could in theory result in the design of a very versatile protective device which could adjust its response depending on the type of collision and hence minimise the damage of the object protected by magneto-mechanical metamaterial.

## Model

### Geometry

**Figure 1:**
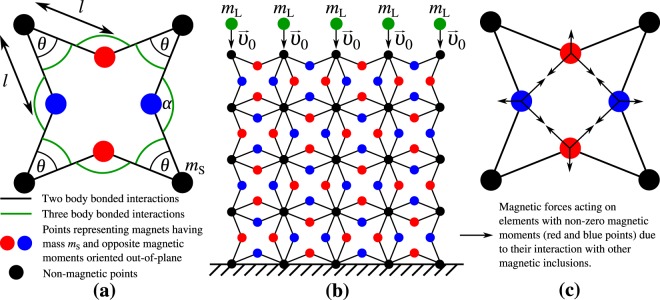
On panel (a) one can see a single unit-cell of the considered system with all of the geometric parameters provided. Panel (b) shows an example of the considered system which is composed of *N*_*x*_ × *N*_*y*_ = 4 × 4 unit-cells (the results are generated for a system composed of a different number of units). Points at the bottom of the structure are fixed to the protected body and cannot move in any direction. The green points above the structure having certain initial velocity induce the deformation of the system as a result of the inelastic collision. On panel (c) one can see magnetic forces acting on magnetic inclusions inserted into the system composed of a single structural unit. All panels show the geometry of the structure before it was subjected to a mechanical deformation.

The design of the mechanical metamaterial investigated in this study was loosely inspired by the work of Grima *et al*.^18^ on star-shaped systems. However, in the case of this study the star-shaped units were directly joint together from their outmost vertices (see Fig. 1) rather than from the vertices at the centre of star-shaped units (through the use of additional ribs) as was the case for Grima *et al*. This change in connectivity alters the way the system deforms when loaded. In fact, contrary to the star-based systems studied by Grima *et al*., the system presented here does not exhibit auxetic behaviour when deformed in the vertical direction through the use of outmost vertices. In addition to that, in a number of cases studied in this work (see section “Types of systems investigated in this work” for a detailed description), magnetic inclusions were also added to the vertices of the star-shaped units in order to control the behaviour of the system through magnetic interactions. It should be also mentioned that the use of a geometry with a positive Poisson’s ratio is deemed to be important as it allows checking whether any enhancement in the indentation resistance is a result of the introduction of magnetic inclusions and their appropriate distribution within the system rather than an effect of the negative Poisson’s ratio of the geometry.

The two-dimensional model which is investigated in this work was designed in a manner allowing it to mimic the behaviour of real systems (see the Results and Discussion section). More specifically, it consists of appropriately distributed points having a mass *m*_*S*_ which interact with each other both by means of bonded (representing actual linkages in the structure) as well as non-bonded interactions (representing magnetic interactions within the structure). This system is shown schematically in Fig. 1 where different lines represent different types of bonded interactions. In terms of geometry, the considered system consists of *N*_*x*_ × *N*_*y*_ star-shaped unit-cells where each of the unit-cells is symmetric with respect to its centre and has four corners. Each unit-cell is represented by means of eight line-segments (solid black lines) having a length of *l* which connect different pairs of magnetic and non-magnetic inclusions having a mass *m*_*S*_ as indicated in Fig. 1. In the case of each of the unit cells, the interior angle between line segments forming the four corners is denoted as *θ*.

### Interactions

As it was already mentioned, one type of interactions between points having a mass *m*_*S*_ which are present in this model are bonded interactions. As a matter of fact, in the considered system there are two types of such interactions. More specifically, two body bonded interactions which are denoted by means of solid black lines connecting different pairs of points and three body bonded interactions corresponding to hinges formed between different pairs of adjacent black lines which are marked by means of green arcs in Fig. 1(a). In this work, the two body bonded interactions are assumed to be governed by the harmonic potential associated with a certain equilibrium length of ligaments *l*_0_. This means that for any situation where the distance *l* between a pair of points would assume a value different than *l*_0_, there would be a non-zero restoring force acting on both of these points (see Methods section). This in turn allows to mimic the behaviour of elastic materials having their components stretched. Furthermore, in this work it is also assumed that three body bonded interactions responsible for hinging of appropriate elements within the system (see Fig. 1(a)) are governed by the harmonic potential for the rotational motion which depends on the deviation in the magnitude of angles *θ* and *α* from equilibrium angles associated with the hinges, i.e. *θ*_0_ and *α*_0_. Such approach allows to represent the hinging process for a variety of materials including perforated elastic structures^61,62^.

In addition to bonded interactions, some of the points constituting the considered system also interact with each other by means of non-bonded interactions. This stems from the fact that it is assumed that some of the points constituting the structure have a magnetic moment $\overrightarrow{\mu }\,(\mu =|\overrightarrow{\mu }|)$ associated with them. It is also assumed that all of the magnetic moments within the system are oriented in the orthogonal direction to the plane formed by the considered system, however the respective magnetic moments can be parallel or antiparallel with respect to each other depending on their position within the system (see Fig. 1). This means that magnetic inclusions located within the same unit primarily attract each other as shown schematically in Fig. 1(c). This in turn makes the compression of the system easier in comparison to the similar non-magnetic structure. This is important as for a majority of materials, soft structures that are easy to compress normally act as better protective devices than their stiffer counterparts. Furthermore, due to the nature of magnetic interactions, each of the inclusions having a non-zero magnetic moment interacts magnetically with all of the other inclusions having a non-zero magnetic moment. It should also be emphasised that the approach described here may correspond to a realistic scenario where one would insert magnetic inclusions, for example in the form of thin cylindrical neodymium magnets, into appropriate positions within the system which are marked by means of red and blue dots in Fig. 1. At this point, it should also be mentioned that the initial configuration of the system (see Methods section for particular parameters) was such that the magnitude of its internal interactions was at its minimum. This in turn leads to the situation where a predominant factor determining the deformation of the considered system is a collision with an external body and not internal bonded and non-bonded interactions. At this point, it should be also mentioned that the evolution of the considered system due to all of the types of interactions present in this model is analysed through numerical Molecular Dynamics simulations^56^ as discussed in more detail in the Methods section.

### Cause of deformation and boundary conditions

To induce a mechanical deformation which mimics an inelastic collision of the considered system with an external body, it is assumed that topmost points within the system have a mass *m*_*L*_, where *m*_*L*_ > *m*_*S*_ (these points are indicated by means of green dots in Fig. 1) and that each of these points has an initial velocity $\overrightarrow{{\upsilon }_{0}}$ directed downwards as shown in Fig. 1(b). Furthermore, in order to prevent the entire structure from shifting in space once it is being hit, it is also assumed that bottommost points within the structure are fixed in space. These assumptions are expected to allow the investigated model to represent the behaviour of realistic systems when subjected to an inelastic collision with a heavy external body.

### Types of systems investigated in this work

**Figure 2:**
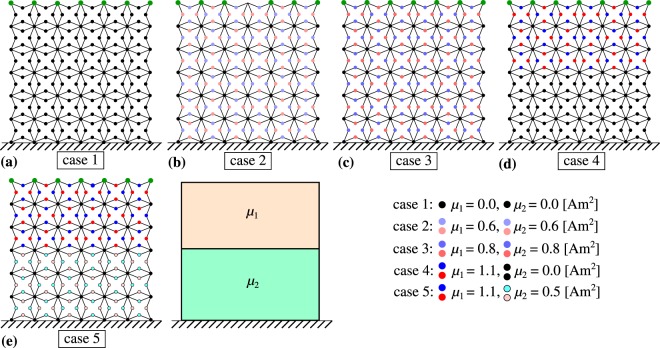
A diagram showing four different examples of the system which were investigated in this work. All of these systems are identical in terms of the initial geometry and mass distribution and the only thing which is different between the considered cases is the magnitude of the magnetic moment associated with magnetic inclusions within each of the systems. For the sake of simplicity, three-body bonded interactions are not indicated in this figure. It is also important to note that results were generated for larger systems than those shown in this figure, i.e. *N*_*x*_ × *N*_*y*_ = 12 × 6.

In order to analyse the potential of the considered system to act as an efficient protective device during the collision with an external body, different configurations of the system were taken into consideration to determine the type of the design which would allow to minimise the damage inflicted on the body protected by the metamaterial. For all of the considered scenarios (see Fig. 2), both the initial geometry and mass distribution within the system were the same which allows to reliably asses the effect which the use of magnetic inclusions has on the behaviour of the system. Also, all of the systems investigated in this work were composed of *N*_*x*_ × *N*_*y*_ = 12 × 6 unit-cells.

The first system which was investigated in this work is shown schematically in Fig. 2(a) (case 1) and consists of inclusions which do not have a magnetic moment, i.e. *μ* = 0 for all of the points. This means that only bonded interactions are present in such system and none of the points attract nor repel each other as a result of magnetic interactions. In the case of the second system which was chosen to be analysed in this work (see Fig. 2(b)), magnetic inclusions located close to the center of respective structural units have a magnetic moment of magnitude 0.6 Am^2^, where such magnetic inclusions are distributed uniformly in the entire system. Thus, one should expect that it would be much simpler to deform the latter system when compared to its non-magnetic counterpart as in this case the attraction of magnetic inclusions promotes the compression of the system. The third case investigated in this work is almost identical to the structure shown in Fig. 2(b) with the only difference being the magnitude of the magnetic moment associated with magnetic inclusions (see Fig. 2(c)).

In addition to the uniform distribution of magnetic inclusions, we also analyse different types of arrangements of magnetic inclusions in an attempt of identifying efficient protective devices. One of such structures that is investigated in this study is shown in Fig. 2(d), where magnetic inclusions corresponding to a magnetic moment of magnitude 1.1 Am^2^ are located only in the top part of the structure. In the bottom part of this structure only non-magnetic inclusions are present which makes the entire system resemble a laminate in terms of the distribution of magnetic inclusions. The last scenario which was investigated in this work is shown schematically in Fig. 2(e) where one can note that similarly to the system shown in 2(d) it is designed in a laminate-like manner. The main difference between these two structures is the fact that the system shown in 2(e) has magnetic inclusions located in its bottom part. However, it should be noted that these inclusions correspond to a smaller magnetic moment than magnetic inclusions located in the top part of the composite.

## Results and Discussion

**Figure 3:**
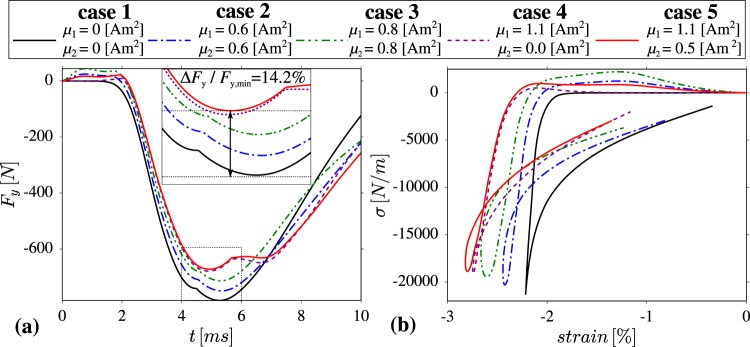
Panels show (a) the maximum vertical force applied to the protected body in time as a result of the collision of an external body with the protective layer of the mechanical metamaterial and (**b**) stress (plotted against the corresponding compressive vertical strain associated with the change in height of the magneto-mechanical metamaterial) induced at the interface between the protected material and the metamaterial as a result of the collision of an external object with the protective layer of the mechanical metamaterial.

In this work, the behaviour of different magnetic and nonmagnetic mechanical metamaterials described in the Model section was analysed in an attempt to determine the arrangement of magnetic inclusions that would allow to increase the impact resistance of a protective material. To do that, we compare the force applied to the protected material at the interface between considered magnetic mechanical metamaterials and the protected object. As shown in Fig. 3(a), depending on the magnitude of the magnetic moment associated with magnetic inclusions and their distribution within the protective mechanical metamaterial, the maximum force (in terms of magnitude) applied to the protected body assumes very different values in time. This is particularly visible at the time when the largest (in terms of magnitude) force is acting on the protected body which for all of the analysed cases happens after approximately 5 ms since the initial collision. According to the provided results, in the case of the nonmagnetic metamaterial (case 1), a larger maximum force is being applied to the protected body than is the case for all of the metamaterials with magnetic inclusions (cases 2–5). This means that the systems with magnetic inclusions considered are better protective devices than their nonmagnetic counterpart as their use leads to a smaller force acting on the protected body.

**Figure 4:**
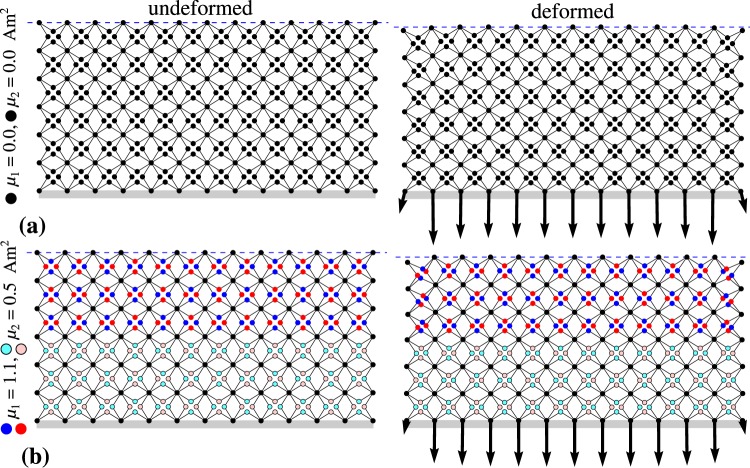
Deformation of two of the considered systems induced by the collision with an external body. These systems are identical in terms of the initial geometry and mass distribution but have different types of magnetic inclusions inserted into them. Panels show, (a) undeformed and deformed configuration assumed by the system composed of nonmagnetic inclusions and (**b**) undeformed and deformed configuration of the magnetic system constructed in a way so that in its top and bottom part magnetic inclusions are different and correspond to the magnetic moment of magnitude 1.1 Am^2^ and 0.5 Am^2^ respectively. In both of these cases, the deformed configuration corresponds to the situation when the largest vertical force is being applied to the protected body. Such forces are graphically indicated by means of black arrows. The horizontal blue dashed line is used to indicate the position of the topmost point within each of the considered systems.

In addition to the above observation, it is also interesting to a analyse the relative quantitative improvement regarding the reduction of the force transmitted to the protected body for different examples of the considered model. More specifically, based on Fig. 3(a) and Table 1, it can be noted that the use of uniformly distributed magnetic inclusions having a magnetic moment 0.6 Am^2^ (case 2) allows to reduce the maximum (in terms of magnitude) force applied to the protected body by 4.35%. Furthermore, for the identical distribution of magnetic inclusions, the increase in the magnetic moment from 0.6 Am^2^ to 0.8 Am^2^ leads to the reduction of the maximum force associated with the nonmagnetic system by 8.97%. This clearly shows that even a relatively small change in the strength of magnetic inclusions may significantly improve protective properties of the magneto-mechanical metamaterial. It should be also noted that in addition to the magnitude of the magnetic moment corresponding to magnetic inclusions, another way to improve the impact resistance of the system corresponds to the appropriate distribution of magnetic inclusions. More specifically, in Fig. 3(a) and Table 1 it is shown that the use of the system that has strong magnetic inclusions in its top part and nonmagnetic inclusions in its bottom part (case 4) results in the application of a smaller force (in terms of magnitude) to the protected body than is the case for the metamaterial with uniformly distributed magnetic inclusions. This is particularly interesting as in the case of the system referred to as case 4, the total magnetic moment associated with all of the magnetic inclusions is smaller than for the system with uniformly distributed magnetic inclusions (case 3). Furthermore, it is also shown that the suitability of the metamaterial with magnetic inclusions located only in its top part to act as a protective device may be further improved should one decide to additionally use weaker magnetic inclusions in the bottom part of the metamaterial albeit to a small extent. In this case, i.e. case 5, the relative improvement in terms of the maximum force applied to the protected body is equal to 14.2% in comparison to the nonmagnetic structure (case 1). These two cases, i.e. cases 1 and 5, that correspond to the nonmagnetic structure and the most efficient of the considered systems with magnetic inclusions are shown graphically in Fig. 4. For each of these systems, forces acting on the interface between the deformed protective metamaterial and the protected body at the moment when the maximum force was being detected during the deformation process are also shown.

**Table 1:** This table shows minimum values of the vertical force *F*_*y,min*_ and minimum stress *σ*_*min*_ acting on the interface between the metamaterial and the protected body for 5 cases of the system considered in this work with the corresponding results being plotted in Fig. 3.

	case 1	case 2	case 3	case 4	case 5	% Improvement: cases 1–5
*F*_*y*,*min*_[N]	−783.6	−749.5	−713.3	−678.8	−672.1	**14.2**
*σ*_*min*_[kN/m]	−21.27	−20.30	−19.44	−19.10	−18.87	**11.3**

In addition to the analysis of the maximum force applied to the protected body, the suitability of the considered magneto-mechanical metamaterials to act as protective devices can be also assessed upon analysing the stress induced at the interface between the protected body and the metamaterial. As shown in Fig. 3(b), in the case of this work, the stress is presented for corresponding values of the vertical strain which was calculated as the change in the distance between topmost and bottommost part of the structure divided over the initial height of the system (engineering stress). Based on the provided results, one can note all of the trends that were observed upon analysing the maximum force transmitted to the protected body can be also observed in the case of the graph shown in Fig. 3(b). This means that these results confirm the potential of magneto-mechanical metamaterials to exhibit an enhanced impact resistance in comparison to their nonmagnetic counterpart. In addition to that, similarly to the formerly conducted analysis, generated results confirm the importance of the distribution of magnetic inclusions as the structure exhibiting the best impact resistance is the one with the non-uniform distribution of magnetic inclusions (case 5). In here, the relative improvement in terms of the applied stress between cases 1 and 5 is equal to 11.3%. At this point, it should be also mentioned that the suitability of the considered magneto-mechanical metamaterials to act as protective devices can also be deduced based on the difference in the extent of the vertical strain which stems from the fact that soft elastic materials are normally expected to exhibit a better impact resistance than their stiffer counterparts. An explicit analysis of the change in height of the system after the collision with an external body is provided in the Supplementary Information.

Before concluding, it should be emphasised that the model discussed in this work may mimic the behaviour of real systems constructed in the appropriate manner. This stems from the fact that for the purpose of the analysis, the ligaments constituting the system were assumed to be relatively stiff so that their length remains approximately constant throughout the entire process of the deformation. Such ligaments were also assumed to have macroscopic linear dimensions, i.e. a length of two centimeters in this particular case (see Methods section). This means that ligaments like that could be easily produced by means of 3D extrusion printers for example through the use of the PLA plastic. The hinging process between ligaments could be invoked by the appropriate design of ends of ligaments in a similar manner to models presented in^29,30^. For such structure, the hinging process could be additionally governed by springs connecting adjacent ligaments in a manner resembling the behaviour of theoretical hinges having their motion governed by the harmonic potential. It should also be noted that similar results to those reported in this work could be expected to be observed for elastic ligaments where such ligaments could be produced for example through the use of the elastic material such as silicon or through the use of an elastic material that can be used with stereolithography 3D printers. Furthermore, the effect which the use of magnetic inclusions has on the behaviour of the model considered in this work could be replicated by the use of appropriately distributed thin cylindrical neodymium magnets with a similar approach being used in already published experimental studies related to magneto-mechanical metamaterials^29,30^. It should be also mentioned that the geometry discussed in this work is just one of many possible examples of mechanical metamaterials that could serve as a host for magnetic inclusions enhancing the impact resistance of the system. In fact, should one consider the use of the non-magnetic structure that has an intrinsic capability to act as a protective device, then the appropriate use of magnetic inclusions could further enhance its impact resistance.

All of this is very important as in this work it is shown that the use of magnetic inclusions may enhance indentation resistance of non-magnetic mechanical metamaterials. It is also shown that through the variation in the magnitude of magnetic moments associated with magnetic inclusions and their distribution, it is possible to control the magnitude of the force applied to the protected body as a result of a collision with an external object, i.e. the cause of the damage inflicted on the protected body. As the matter of fact, these results suggest that it should be possible to design programmable impact resistant devices where magnetic inclusions discussed in this work could be replaced by electromagnets. As a result, one could easily control the strength of magnetic interactions via the magnitude of the current which in turn could allow such devices to act differently depending on the type of the collision. In the case of standard magnetic inclusions, a similar effect could also be achieved upon replacing one type of magnetic inclusions with another set of inclusions which process does not require the reconstruction of the entire system as would normally be the case for conventional non-magnetic protective devices in order to observe such programmable behaviour. This offers a significant advantage in comparison to standard protective devices as the response of the mechanical metamaterial could be conveniently fine-tuned. All of this indicates that the use of magnetic inclusions could lead to the design of superior protective devices as they could not only further enhance the efficiency of already existing and similar protective devices but also make it simple to adjust the type of the response of the protective material to a particular situation with a similar process being very cumbersome for conventional protective materials.

At this point, it should also be highlighted that even though in this work a non-auxetic system was taken into consideration to asses the potential of magnetic inclusions to induce the enhanced impact resistance it does not mean that this concept cannot be extended to auxetic and other systems which posses an intrinsic indentation resistance. As the matter of fact, it should be expected that the use of appropriately distributed magnetic inclusions should further increase the capability of auxetic systems to protect a given object from the damage inflicted on it by the collision. This also means that one can use magnetic inclusions and results reported in this work to increase efficiency of already existing protective devices including military-related applications and car bumpers where the need for materials exhibiting properties reported in this work is commonly known.

## Conclusions

In this work, through numerical studies, it was shown that mechanical metamaterials with magnetic inclusions can exhibit an enhanced impact resistance in comparison to their non-magnetic counterparts. It was also shown that upon increasing the magnitude of the magnetic moment associated with magnetic inclusions within the system the impact resistance of the entire structure gets improved. Another interesting result reported in this work corresponds to the effect which the distribution of magnetic inclusions within the system has on the capability of the structure to minimise the negative effects of the collision with an external body. More specifically, it was shown that through an appropriate design of the considered composite system, it is possible to minimise the force applied to the protected body as a result of the collision. It can be also concluded that the concept reported in this work can be used in order to improve the efficiency of the already existing protective devices and thus is expected to prove to be useful in the case of a variety of applications ranging from military-related protective devices to devices which we could use in our everyday lives such as car bumpers.

## Methods

This work is a numerical study making use of Molecular Dynamics simulations meant to asses the suitability of systems described in the Model section to act as protective devices in the case of the collision with an external body. To do this, differential equations taking into account all of the interactions present in this system are solved numerically by means of the fourth-order Runge-Kutta method^63^ with a constant time step Δ*t* = 10^−6^ s and the corresponding error of the order *O*(*h*^5^). It should be also noted that selected parameters allow to reliably simulate the behaviour of the considered system.

As it was mentioned in the Model section, points constituting the considered system interact with each other both by means of bonded and non-bonded interactions (see Supplementary Information). Two body bonded interactions, that describe the mutual attraction or repulsion between a pair of points, allow to mimic the behaviour of a ligament in the real-life experimental system that is subjected to a longitudinal deformation. However, as it was already mentioned in the Model section, in this work, the stiffness corresponding to ligaments was set to be relatively high so that the length of ligaments would remain approximately the same throughout the entire deformation. As a result, the primary mechanism governing the deformation of the considered system is the hinging between different pairs of adjacent bonds that are connected to each other at a single point. Such hinges are formed by means of different sets of three points as shown schematically in Fig. 1(a). In this work, it is assumed that both the two body and the three body bonded interactions are governed by means of appropriate harmonic potentials as described below. More specifically, the potential governing two body bonded interactions^32,56,64^ and the magnitude of the resulting force acting on respective points connected by a ligament having of length *l* can be described in the following manner:

1 $${V}_{bonded2}=\frac{1}{2}k{(l-{l}_{0})}^{2}\,\,\,\,\,F=\mp \,k(l-{l}_{0})\mp {C}_{1}\upsilon (t)$$

where, *k* stands for a stiffness constant. The second term in the expression describing *F* is responsible for the damping of the translational motion of points connected by the bond. It was added to make the system more realistic with a similar approach being commonly utilised in other computational studies involving mechanical systems^7^. In here, *C*_1_ is the damping constant and *υ*(*t*) is the difference of linear velocities of two points connected by the same bond at time *t* (see Supplementary Information to see the way how damping affects the energy of the system in time). Furthermore, the three-body bonded interactions^64^ were also assumed to be governed by means of the harmonic potential for the rotational motion which can be defined as follows:

2 $${V}_{bonded3}=\frac{1}{2}{K}_{h}{(\theta -{\theta }_{0})}^{2}\,\,{\rm{or}}\,\,{V}_{bonded3}=\frac{1}{2}{K}_{h}{(\alpha -{\alpha }_{0})}^{2}\,\,{\rm{or}}\,\,{V}_{bonded3}=\frac{1}{2}{K}_{h}{(\gamma -{\gamma }_{0})}^{2}$$

depending on which hinge is taken into consideration. In this case, *K*_*h*_ stands for the stiffness constant for the rotational motion. Angle *γ* is the angle between adjacent ligaments from two neighbouring star-shaped structural units (see Supplementary Information). For such potential, the torque that was chosen in this work to be responsible for the motion of points constituting the hinges was defined as follows:

3 $$\tau =\mp \,{K}_{h}({\rm{angle}}-{{\rm{angle}}}_{eq})\mp {C}_{2}\omega (t)$$

where angle and angle_*eq*_ can replaced by any of aforementioned variables depending on the type of the hinge, e.g. *θ* and *θ*_0_. In here, the second term is responsible for the damping of the hinging motion which allows to produce realistic results^7^. More specifically, *C*_2_ is a damping constant and *ω*(*t*) is the rate of change in the value of the given angle at a specific time *t*.

In addition to the bonded interactions, the only interactions which are present in the system correspond to the magnetic attraction/repulsion between magnetic inclusions. The force of the mutual interaction experienced by magnetic moment *i* as a result of the interaction with magnetic moment *j* can be defined in the following manner^65^:

4 $$\overrightarrow{{F}_{ij}}=\frac{3{\mu }_{0}}{4\pi {r}^{5}}(\overrightarrow{{\mu }_{i}}\cdot \overrightarrow{{\mu }_{j}})\overrightarrow{r}$$

where, *μ*_0_ is the vacuum permeability and *r* is the distance between the *i*-th and *j*-th magnetic moment. In here, it is assumed that magnets may interact with each other as long as the distance between their centres *d*_*MAG*_ > 0.5 cm. Once the value of *d*_*MAG*_ reaches 0.5 cm, the elastic collision between the two inclusions is observed. This condition allows for more realistic simulations where magnetic inclusions that in reality could be represented by neodymium magnets having finite non-zero dimensions (diameter of 5 mm for the considered value of *d*_*MAG*_). Furthermore, it should be mentioned that all of the parameters used in this work were set to be the following: *l*_0_ = 2 cm, *l*(*t* = 0) = 2 cm, *θ*_0_ = 26.0°, *θ*(*t* = 0) = 26.0°, *α*_0_ = 116.0°, *α*(*t* = 0) = 116.0°, *γ*_0_ = 64°, *γ*(*t* = 0) = 64°, *m*_*S*_ = 50 g, *m*_*L*_ = 250 g, *υ*_0_ = 6 ms^−1^, *k* = 10^6^ k gs^−2^, *K*_*h*_ = 15 J rad^−2^, *C*_1_ = 25 kg s^−1^, *C*_2_ = 25.0 kgm^2^s^−1^.

## Supplementary information

Supplementary Information

## Data Availability

The datasets generated during and/or analysed during the current study are available from the corresponding author on reasonable request.
