# Supplementary material for: Impact resistance of composite magnetic metamaterials
Source: Sci Rep. 2019 Mar 8;9:3963. doi: 10.1038/s41598-019-40610-w (PMC6408472; doi:10.1038/s41598-019-40610-w)
Supplement: Supplementary file 1 — Supplementary Information [file 41598_2019_40610_MOESM1_ESM.pdf]

# Supplementary Information

Impact resistance of composite magnetic metamaterials

K. K. Dudek*[1,2], W. Wolak[1], R. Gatt[2], and J. N. Grima[2,3]

[1]Institute of Physics, University of Zielona Gora, ul. Szafrana 4a, 65-069 Zielona Gora, Poland
[2]Metamaterials Unit, Faculty of Science, University of Malta, Msida MSD 2080, Malta
[3]Department of Chemistry, Faculty of Science, University of Malta, Msida MSD 2080, Malta

# 1 Dissipation of energy

As it was discussed in the main text, in order to produce more realistic results, it was assumed that bonded interactions governing the motion of points constituting the system are being damped. As a result, the total energy of the entire system must decrease in time which behaviour is normally observed in experiments. In order to better visualise how the damping affected the total energy $E$ of each of the systems analysed in the main text, plots showing the variation of energy with time were produced as shown in Fig. S 1. Based on these results, one may note that irrespective of the type of the analysed system, a very similar behaviour in terms of the variation in the total energy may be observed, i.e. the energy $E$ decreases in time at an increasingly slower rate. It should be also emphasised that the obtained trends are very similar to those observed in the case of other studies related to impact resistance of mechanical metamaterials (see the main text for references).

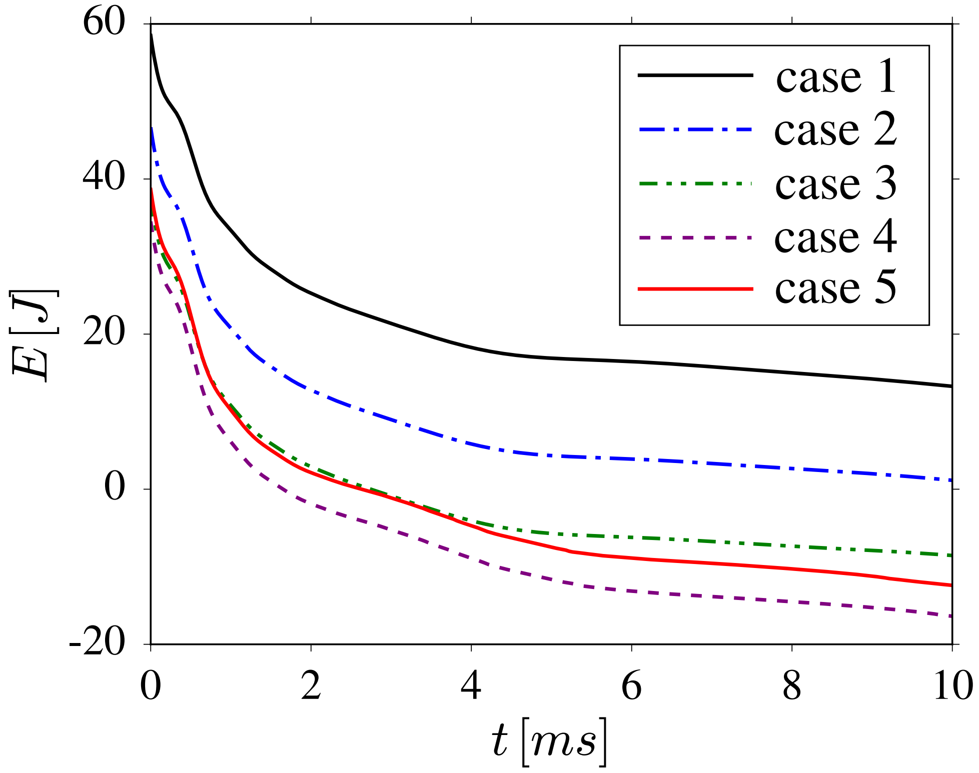


FIG. S 1: The figure shows the variation in the total energy of the system $E$ in time for each of the systems analysed in the main text. In here, the respective cases are denoted in the same manner as it was the case in the main text, i.e. case 1: $\mu_1 = \mu_2 = 0\,[\mathrm{Am}^2]$, case 2: $\mu_1 = \mu_2 = 0.6\,[\mathrm{Am}^2]$, case 3: $\mu_1 = \mu_2 = 0.8\,[\mathrm{Am}^2]$, case 4: $\mu_1 = 1.1, \mu_2 = 0.0\,[\mathrm{Am}^2]$ and case 5: $\mu_1 = 1.1, \mu_2 = 0.5\,[\mathrm{Am}^2]$.

# 2 Variation in height of the system

In addition to the direct analysis of the force acting on the protected body as a result of the collision with an external object, the suitability of a given metamaterial as a protective shielding device may also be analysed based on its macroscopic behaviour upon being subjected to an impact. It seems that one of the most reasonable parameters to do that is the height of the structure. In the case of this work, this quantity was measured as a vertical distance between topmost and bottommost points constituting the material.

It is commonly established that normally softer materials tend to absorb the impact with an external body better than their stiffer counterparts which often makes them act better as protective devices. Based on Fig. S 2, one can note that the height of the nonmagnetic system changed to a smaller extent than was the case for magnetic structures. This in turn is in agreement with results reported in the main text where it was shown that the use of such systems results in the application of a smaller force (in terms of magnitude) to the protected body.

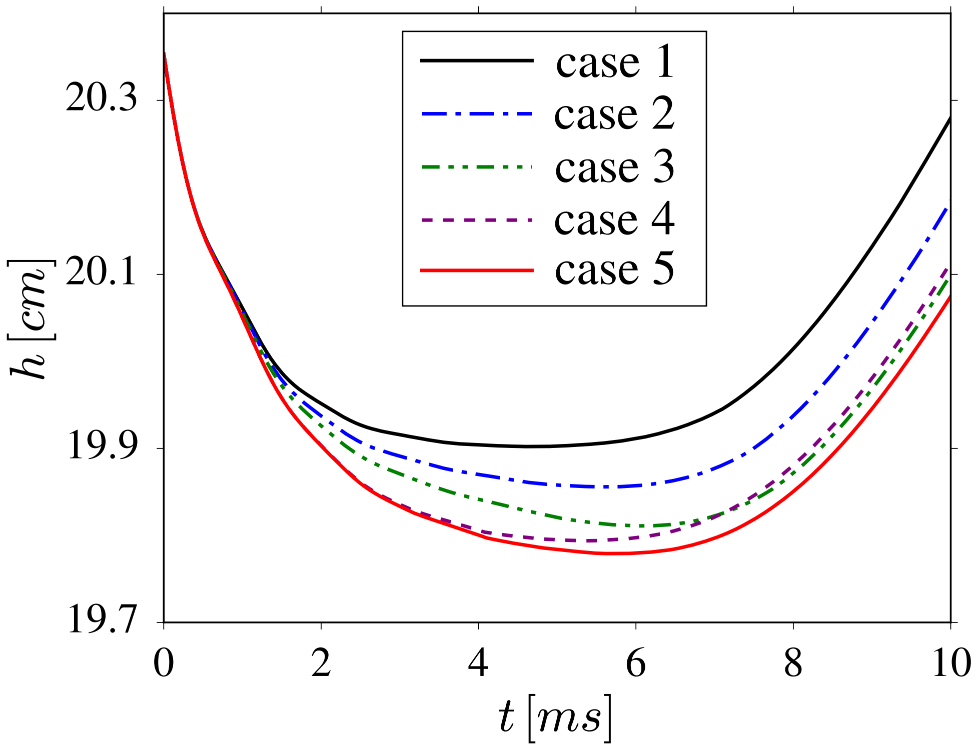


FIG. S 2: The figure shows the variation in height of the structure $h$ in time for each of the systems analysed in the main text. In here, the respective cases are denoted in the same manner as it was the case in the main text, i.e. case 1: $\mu_1 = \mu_2 = 0\,[\mathrm{Am}^2]$, case 2: $\mu_1 = \mu_2 = 0.6\,[\mathrm{Am}^2]$, case 3: $\mu_1 = \mu_2 = 0.8\,[\mathrm{Am}^2]$, case 4: $\mu_1 = 1.1, \mu_2 = 0.0\,[\mathrm{Am}^2]$ and case 5: $\mu_1 = 1.1, \mu_2 = 0.5\,[\mathrm{Am}^2]$.

# 3 Bonded interactions

Bonded interactions that are present in the considered system are defined by means of appropriate equations in the Methods section in the main text. However, to better understand how the use of two-body and three-body bonded interactions affects inclusions corresponding to a given type of the interaction, Fig. S3 and Fig. S4 associated with the two-body and three-body bonded interactions are provided. It is important to note that in each of the considered cases, the sum of all of the force must be equal to zero so that conservation laws would be satisfied. Hence, in the case of three-body interactions, $\vec{F_1} + \vec{F_2} + \vec{F_3} = \vec{0}$.

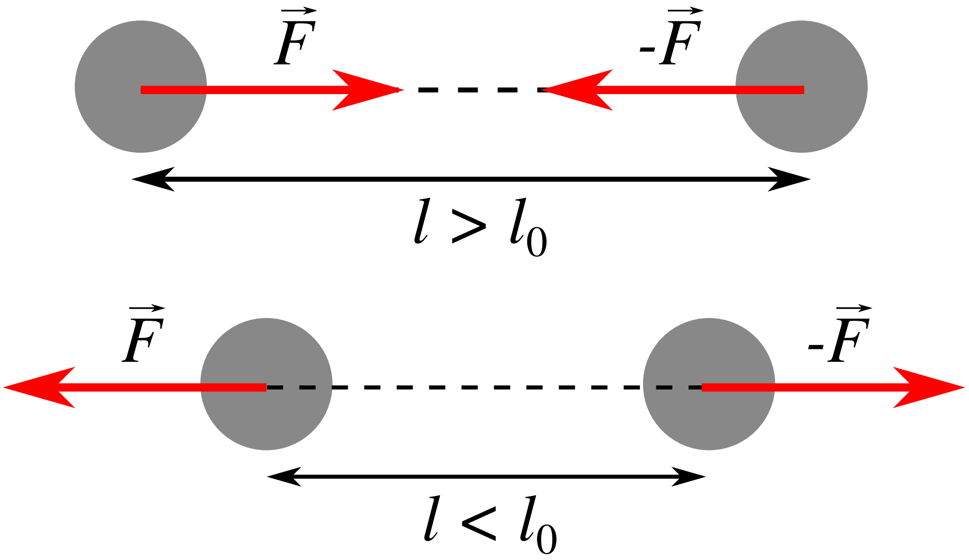


FIG. S 3: Graphical representation of forces acting on a pair of points as a result of the two-body interaction.

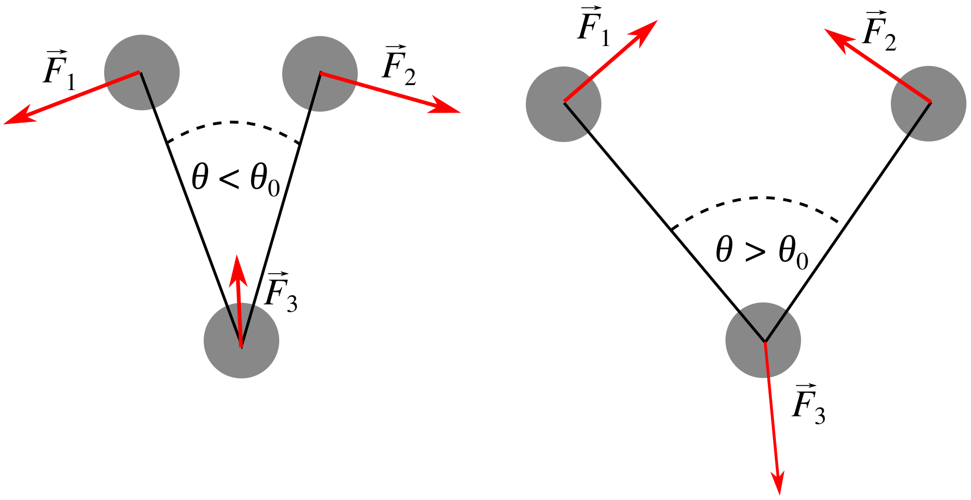


FIG. S 4: Graphical representation of forces acting on a set of three points as a result of the three-body interaction.

## 4 Three-body bonded interactions within the structure

In order to better indicate the which of the points within the system interact with each other by means of the three-body bonded interactions, Fig. S.5 was prepared where such interactions are graphically represented by means of solid green lines.

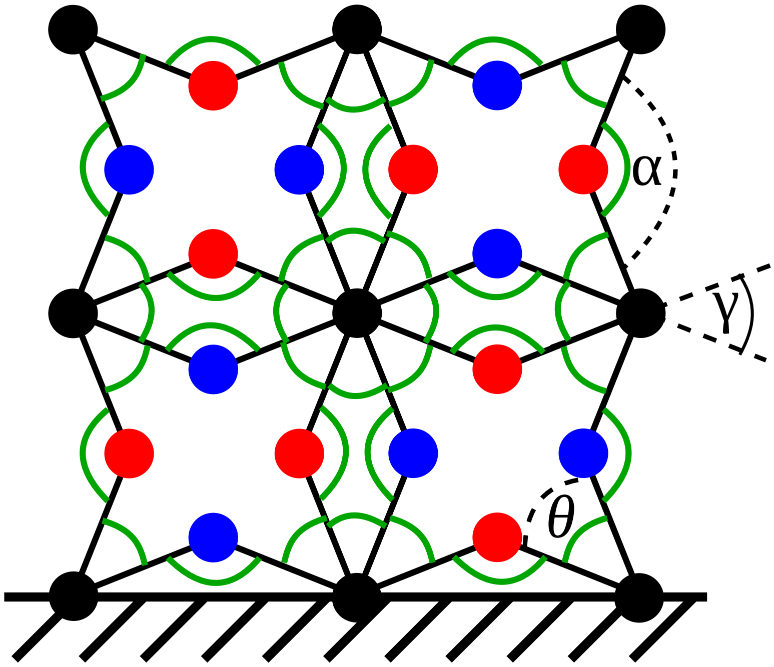


FIG. S 5: Graphical representation of three-body bonded interaction within the system composed of $2 \times 2$ structural units. In this case, three-body interactions are marked by means of solid green lines.
